# Characterization of the malaria parasite *Plasmodium falciparum* Tepsin homolog

**DOI:** 10.1128/spectrum.03288-24

**Published:** 2025-06-30

**Authors:** Stéphanie Roucheray, Maria R. Narciso, Dave Richard

**Affiliations:** 1 Department of Microbiology-Infectious Diseases and Immunology, Faculty of Medicine, Université Laval, Quebec City, Canada; 2 CHU de Québec-Université Laval Research Centre, Québec, Canada; 3 Centre de Recherche en Infectiologie de l’Université Laval, Quebec City, Canada; 4 Department of Chemistry and Biology, Molecular Science Graduate Program, Toronto Metropolitan University, Toronto, Canada; Weill Cornell Medicine, New York, New York, USA

**Keywords:** malaria, protein trafficking, Golgi, Tepsin

## Abstract

*Plasmodium* parasites rely on the invasion of human erythrocytes for their survival. This invasion process is facilitated by specialized organelles (rhoptry, micronemes, and dense granules) housed within a distinctive structure known as the apical complex. How the apical complex is generated is still enigmatic, especially how specificity is achieved in the vesicular trafficking between the Golgi apparatus and the apical organelles, but phosphoinositide lipids might potentially be involved. Here, we describe the characterization of a putative phosphoinositide-binding protein containing an Epsin NH_2_-terminal homology (ENTH) domain, Pf3D7_1459600. We show that this protein is structurally homologous to human Tepsin. Surprisingly, unlike other Tepsins, the ENTH domain of PfTepsin binds non-specifically to phosphoinositides *in vitro*, potentially through a positively charged pocket. Colocalization assays revealed that PfTepsin potentially transits between the Golgi apparatus and some of the apical organelles in developing schizonts. Finally, we provide evidence that PfTepsin potentially interacts with members of the clathrin and adaptor protein 4 complexes.

**IMPORTANCE:**

Malaria takes an enormous toll on affected societies, and new drugs are urgently required. Understanding how the parasite causing malaria replicates could lead to potential new drug targets. Our work characterizes a protein called Tepsin that could potentially be important for the parasite to generate organelles critical for its survival.

## INTRODUCTION

Though considerable investments over the past 20 years have helped in reducing the burden of malaria, it still remains one of the deadliest infectious diseases. In 2023, 263 million cases and 597,000 deaths, most of which were children under 5 years old, were recorded (1). Increasing resistance to most available antimalarials, including artemisinin, highlights the need for new intervention measures (2, 3).

*Plasmodium* spp. are obligate intracellular parasites that belong to the phylum Apicomplexa. Essential to their survival is the invasion of a red blood cell, a multistep process where secretory organelles named micronemes, rhoptries, and dense granules are sequentially discharged (reviewed in reference 4). Though substantial progress has been made in defining the molecular mechanisms underlying the invasion process, much less is known with regard to how the organelles of the apical complex are generated. Several of the parasite’s organelles, such as the Golgi apparatus, the rhoptries, and the inner membrane complex (IMC), form around an outer centriolar plaque to which they remain associated until disappearance late in the merozoite formation process (5). Nascent rhoptry bulbs are first visible in early schizogony, before merozoite segmentation, followed by elongation of the rhoptry neck (5–7). Then, subsets of micronemes containing the apical membrane antigen-1 appear, followed by ones containing erythrocyte-binding antigen 175 (5), consistent with micronemes being heterogenous (8). Intriguingly, conditionally knocking down the Golgi protein PfSortilin was shown to result in the retention of rhoptry, microneme, and dense granule proteins in the endoplasmic reticulum or their mistrafficking to the parasitophorous vacuole (9, 10). This leads to the question as to how the specificity of transport is generated between the Golgi apparatus and the apical organelles.

Phosphoinositides (PIPs) are minor constituent lipids of intracellular membranes in eukaryotic cells, where they play roles in processes such as protein trafficking, the cell cycle, and cytoskeleton dynamics (11–14). They consist of an inositol head group that can be reversibly phosphorylated at positions 3, 4, and 5 through the action of kinases and phosphatases, resulting in seven isoforms: PI3P, PI4P, PI5P, PI(3,5)P2, PI(3,4)P2, PI(4,5)P2, and PI(3,4,5)P3 (13). PIPs are not uniformly distributed in cell membranes but instead are enriched in specific organelles and membrane microdomains. For example, PI4P is highly enriched at the Golgi (15), while PI3P is found in early endosomes in mammalian cells (16). This code is then read by proteins possessing PIP-binding domains with varying binding specificities (17). For example, the phagocytic oxidase domain of p40^phox^ binds specifically to PI3P, while the pleckstrin homology domain of PI-phospholipase C delta recognizes PI(4,5)P2 (18). Other PIP-binding domains are more relaxed in their binding specificity (17).

As in model eukaryotic organisms, PIPs are present in *P. falciparum*, localize to specific cellular compartments (19, 20), and have been shown to play critical roles in the parasite (reviewed in references 21 and 22). For example, PI3P is important in the trafficking of host-cell cytosol-filled vesicles to the digestive vacuole membrane (19, 20, 23–26), in apicoplast biogenesis (27), in the stabilization of the DV under heat stress (28), and in DV dynamics (29). PI4P is required for membrane ingression during the production of individual merozoites at the schizont stage (30). Critically, the *P. falciparum* PI4KIIIb is a target of new classes of antimalarials currently in clinical development (30–35).

Epsin NH_2_-terminal homology (ENTH)-domain proteins belong mainly to the Epsin protein family. In model eukaryotic organisms, Epsin proteins are involved in clathrin-mediated protein trafficking and endocytosis by binding to membranes enriched in PI(4,5)P2 (36). This binding modifies the membrane curvature to facilitate the formation of clathrin-coated invaginations (37–39). In addition to lipids, Epsin proteins also interact with various proteins including clathrin, adaptor complex proteins AP-1 and AP-2, and Eps15 (39). *P. falciparum* possesses an Epsin homolog that, in addition to the ENTH domain, contains some consensus clathrin-binding motifs, a putative PIP-binding site, and dileucine motifs for potential interaction with adaptor protein complexes (40). However, though host-cell cytosol endocytosis is dependent on AP-2 in *P. falciparum*, clathrin is not involved (23, 41), which suggests that PfEpsin likely has other functions. Interestingly, AP-1 was shown to partially colocalize with the rhoptry protein RAP1, suggesting a potential role in the biogenesis of this organelle (42), so perhaps PfEpsin could also be involved in this process.

In an attempt to further delve into the roles of PIPs in *P. falciparum* erythrocytic stage biology, we previously performed a small-scale gene inactivation screen on 24 putative PIP-binding proteins and PIP kinases and PIP phosphatases, where we found 20 genes that could not be inactivated and are therefore potentially essential for the *in vitro* growth of the parasite and 5 genes that were not essential (knockout successfully generated) (24). In the current paper, we will describe our characterization of Pf3D7_1459600, a protein with a putative ENTH domain for which we could not obtain a knockout line. Our results show that the protein has structural homology to human Tepsin, a protein that does not bind PIPs. However, we show that the recombinant PfTepsin ENTH domain has the capacity to bind to several species of PIPs, potentially due to the presence of a positively charged pocket. Localization analyses revealed that PfTepsin is found at the Golgi apparatus and the micronemes during schizont development. Finally, proteomics analysis showed that clathrin and adaptor protein 4 (AP-4) complexes are potential interactors of PfTepsin. Taken together, these results might suggest a potential role for PfTepsin in vesicular trafficking between the Golgi apparatus and the apical complex.

## RESULTS AND DISCUSSION

### Pf3D7_1459600 is a Tepsin homolog

**Fig 1:**
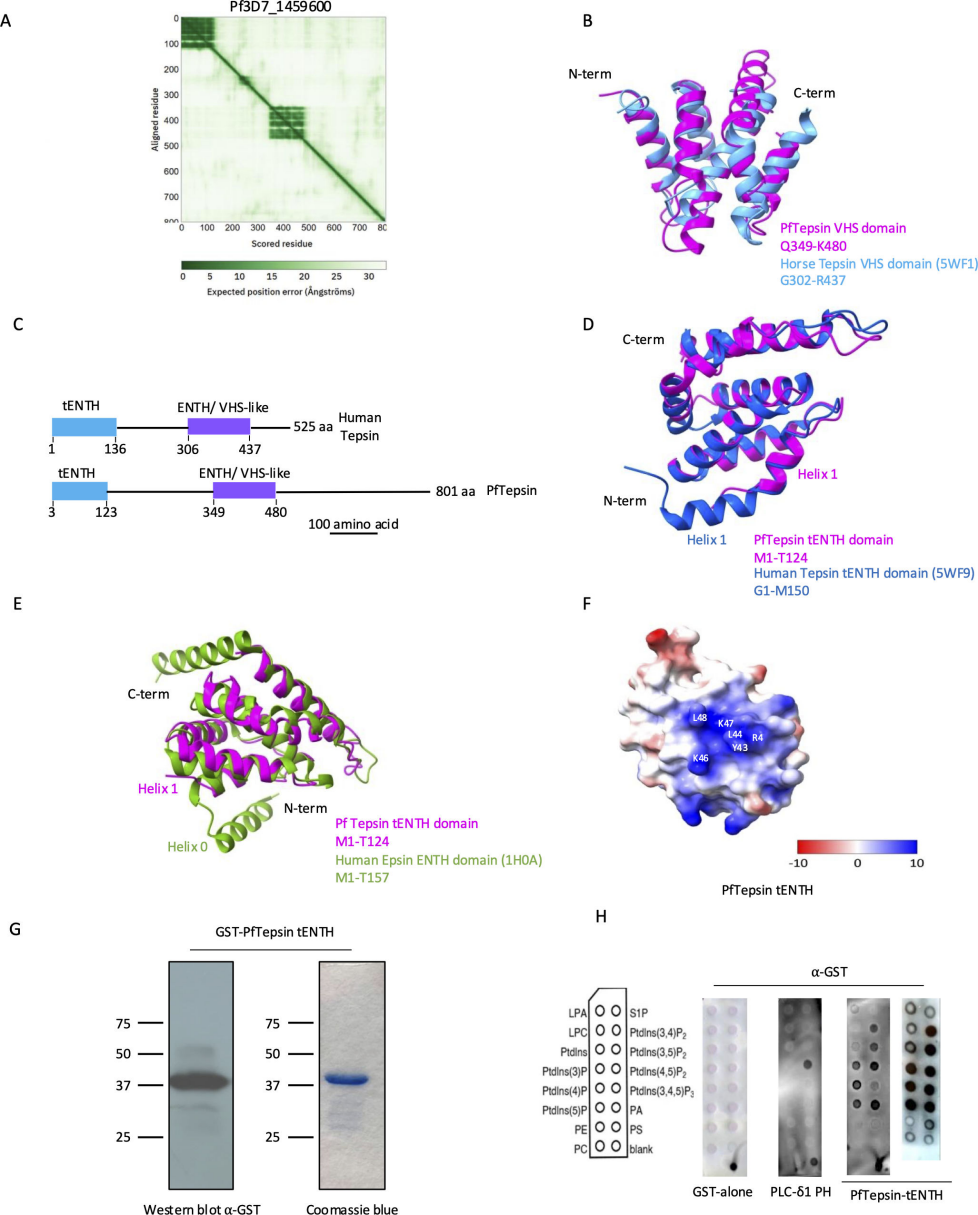
Pf3D7_1459600 is a Tepsin homolog. (**A**) Plot of the predicted aligned error from AlphaFold3 showing two potential domains in Pf3D7_1459600. (**B**) Superposition of the AlphaFold3 structure prediction of the tVHS domain of PfTepsin in pink and the experimentally determined structure of horse Tepsin tVHS domain in blue. (**C**) Domain architecture of PfTepsin and human Tepsin. The tENTH domains are in blue and ENTH/VHS-like domains are in purple. (**D**) Superposition of the AlphaFold3 structure prediction of the tENTH domain of PfTepsin in pink and the experimentally determined structure of human Tepsin tENTH domain in blue. (**E**) Superposition of the AlphaFold3 structure prediction of the tENTH domain of PfTepsin in pink and the experimentally determined structure of the human Epsin ENTH domain in green, showing the absence of the helix α0 in the former. (**F**) Electrostatic surface view of the PfTepsin tENTH domain showing the potential positively charged binding pocket. Scale from negatively charged (red) to positively charged (blue). (**G**) Anti-GST Western blot and Coomassie staining showing the purification of the recombinant GST-PfTepsin tENTH domain. (**H**) Lipid blot showing that the GST-PfTepsin tENTH domain binds to all the species of PIPs and phosphatidic acid. GST alone and the PH domain of phospholipase C-delta1 (binder of PI (4, 5)P2) are used as negative and positive controls, respectively. The signal in the bottom right corner of the membranes is the recombinant proteins, which were spotted as positive controls for the anti-GST antibody.

In the PlasmoDB database (https://plasmodb.org/plasmo/app) (43), the only domain annotated for Pf3D7_1459600 is an ENTH domain at its N-terminus. However, when we inspected the predicted structure of Pf3D7_1459600 in AlphaFold3 (44), a potential additional folded domain with a low predicted aligned error was found from amino acids (AA) 349–480 (Fig. 1A). This particular architecture of an N-terminal ENTH domain followed by a second domain is found in proteins called Tepsins, accessory trafficking proteins of the AP-4 complex (45). In Tepsins, the second domain is called tVHS (Vps27, Hrs, Stam)/ENTH-like (45). To determine if the additional domain in Pf3D7_1459600 was a tVHS domain, we compared its predicted structure with the experimentally determined structure of the horse tVHS domain (Protein Data Bank [pdb]/: 5WF1) (46) and obtained good overlap (root mean square deviation [rmsd] of 1.188 Å), suggesting that Pf3D7_1459600 might be a potential Tepsin homolog (Fig. 1B). We will therefore refer to it as PfTepsin for the remainder of the paper. When we looked in more detail at the architecture of *P. falciparum* and human Tepsins, we found several differences (Fig. 1C). PfTepsin is significantly larger, exceeding human Tepsin by 276 AA. The individual domains in PfTepsin are slightly shorter, with the tENTH domain comprising 123 AA and the ENTH/VHS-like domain spanning 122 AA, compared to human Tepsin with domain lengths of 136 and 131 AA, respectively. Furthermore, the interdomain region is more extensive in PfTepsin, spanning 226 AA, in contrast to the 170 AA observed in human Tepsin (Fig. 1C). We next undertook a structural comparison of the PfTepsin tENTH domain, predicted with AlphaFold3 (44), with the human Tepsin tENTH domain experimentally determined (pdb: 5WF9) (46). Like the human Tepsin tENTH domain, the PfTepsin tENTH domain possesses seven α-helices, and these superpose well with their human counterparts (rmsd of 0.703 Å), though the helix α1 in the N-term of PfTepsin tENTH is shorter (Fig. 1D). Taken together, this analysis suggests that Pf3D7_1459600 is potentially a homolog of Tepsins. In addition, while we were completing this paper, we noticed that Pf3D7_1459600 was now annotated as a putative Tepsin in the PlasmoDB database, supporting our conclusions.

The binding of the ENTH domain of Epsins to PIPs leads to the insertion of an amphipathic helix (referred to as helix α0) into the membrane, resulting in deformation (38, 47). Tepsins, on the other hand, do not possess a helix α0 and require binding to AP-4 to be recruited to membranes (46). Comparison of the structures of the human Epsin ENTH (pdb: 1H0A) (38) and the predicted *P. falciparum* Tepsin tENTH (rmsd of 1.496 Å) showed that the latter indeed lacked the N-term helix-0 (Fig. 1E). Interestingly, closer inspection of the predicted structure of the PfTepsin tENTH domain revealed the presence of a putative positively charged electrostatic surface, which is not usually present in tENTH domains of Tepsins (Fig. 1F) but is similar to what is found in PIP-binding ENTH domains. To determine if the PfTepsin tENTH domain could bind to PIPs *in vitro*, we recombinantly expressed it in fusion with a glutathione-S-transferase tag. An anti-GST Western blot and Coomassie staining on the purified protein fraction revealed a band at the expected size at 37 kDa (Fig. 1G). To assess its PIP-binding capacity, we incubated GST-PfTepsin tENTH with PIP-strips, a nitrocellulose membrane spotted with 15 lipids (48). GST alone was used as a negative control, and the PI(4,5)P2-binding pleckstrin homology domain of phospholipase C-δ1 fused to GST was the positive control (18). Unexpectedly, in both biological replicates, the tENTH domain of PfTepsin was able to bind all species of PIPs along with phosphatidic acid (Fig. 1H). This unspecific binding could potentially be due to the positively charged electrostatic surface interacting with the negatively charged headgroups of the PIPs, as seen in actin-binding proteins, for example (49) (Fig. 1F). Whether this truly occurs in a cellular context remains, however, to be investigated since PIP strips do not recapitulate a membrane bilayer.

### Analysis of the expression of PfTepsin across the asexual erythrocytic stages

**Fig 2:**
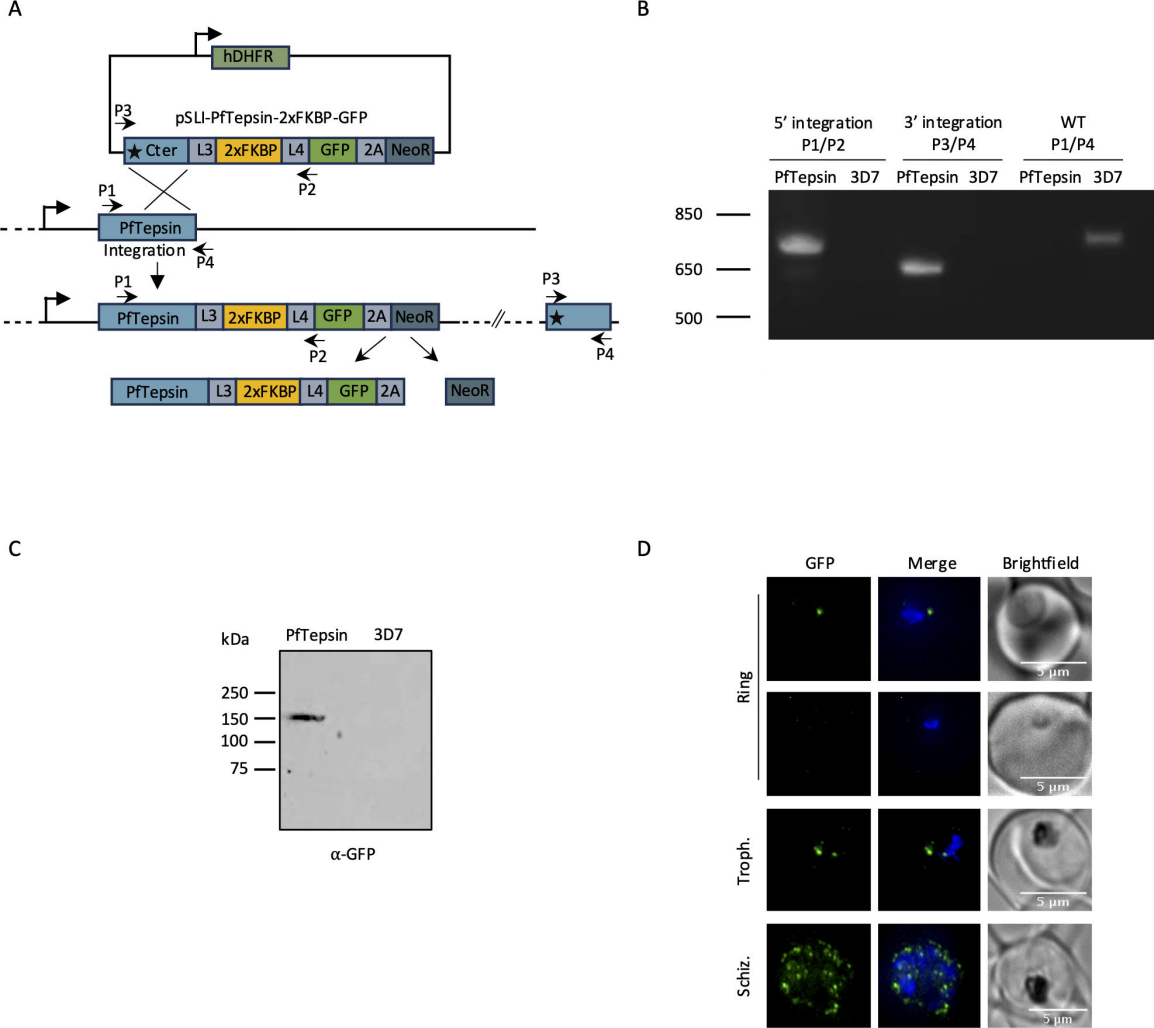
PfTepsin is expressed throughout the asexual erythrocytic cycle. (**A**) Schematic showing the tagging strategy by single cross-over recombination using SLI. (**B**) PCR on parasite genomic DNA showing the proper integration of the tagging vector at the PfTepsin locus (5′ junction: primers P1 and P2, 3′ junction: primers P3 and P4) and the disappearance of the WT allele in the PfTepsin-2xFKBP-GFP line (primers P1 and P4). (**C**) Western blot on mixed-stage parasite protein extracts showing the expression of PfTepsin-2xFKBP-GFP at the expected size of around 149 kDa. (**D**) PfTepsin-2xFKBP-GFP is expressed throughout the asexual erythrocytic cycle. Scale bar represents 5 µm. Blue denotes the 4′,6-diamidino-2-phenylindole (DAPI)-stained nucleus.

To characterize the putative *P. falciparum* Tepsin homolog in parasites, we used the selection-linked integration (SLI) strategy to endogenously tag the C-terminus with GFP by single cross-over recombination (50). To allow the functional analysis of PfTepsin by knock-sideways and the identification of proximal proteins by induced proximity-dependent biotin identification (Di-BioID) (23), a double FK506 binding protein domain (2xFKBP) tag was also appended (Fig. 2A). The correct integration of the vector and the absence of a wild-type (WT) allele were verified by polymerase chain reaction (PCR), demonstrating that we successfully tagged the PfTepsin gene (Fig. 2B). To verify the proper expression of the PfTepsin-2xFKBP-GFP, a Western blot using an anti-GFP antibody on parasite protein extracts taken on an asynchronous parasite culture was performed and revealed a single band at the expected size of around 149 kDa that was absent in the untagged 3D7 control (Fig. 2C). Live microscopy of PfTepsin-2xFKBP-GFP parasites throughout the asexual erythrocytic cycle revealed that the protein was expressed in all observed trophozoites and schizonts, but the signal was often weak in ring stages, and for any experiment, around a third of them did not have a fluorescence signal. In trophozoites and schizont stages, all the observed cells exhibited strong fluorescence. One focus was seen in most rings that had detectable fluorescence, though some seemed to have additional signals. Since the fluorescence was weak, it is hard to tell whether this was background fluorescence. Some trophozoites also had single foci, while others had two foci or more. Multiple foci were found in schizonts (Fig. 2D; Fig. S1).

### Subcellular localization of PfTepsin in schizont stage parasites

**Fig 3:**
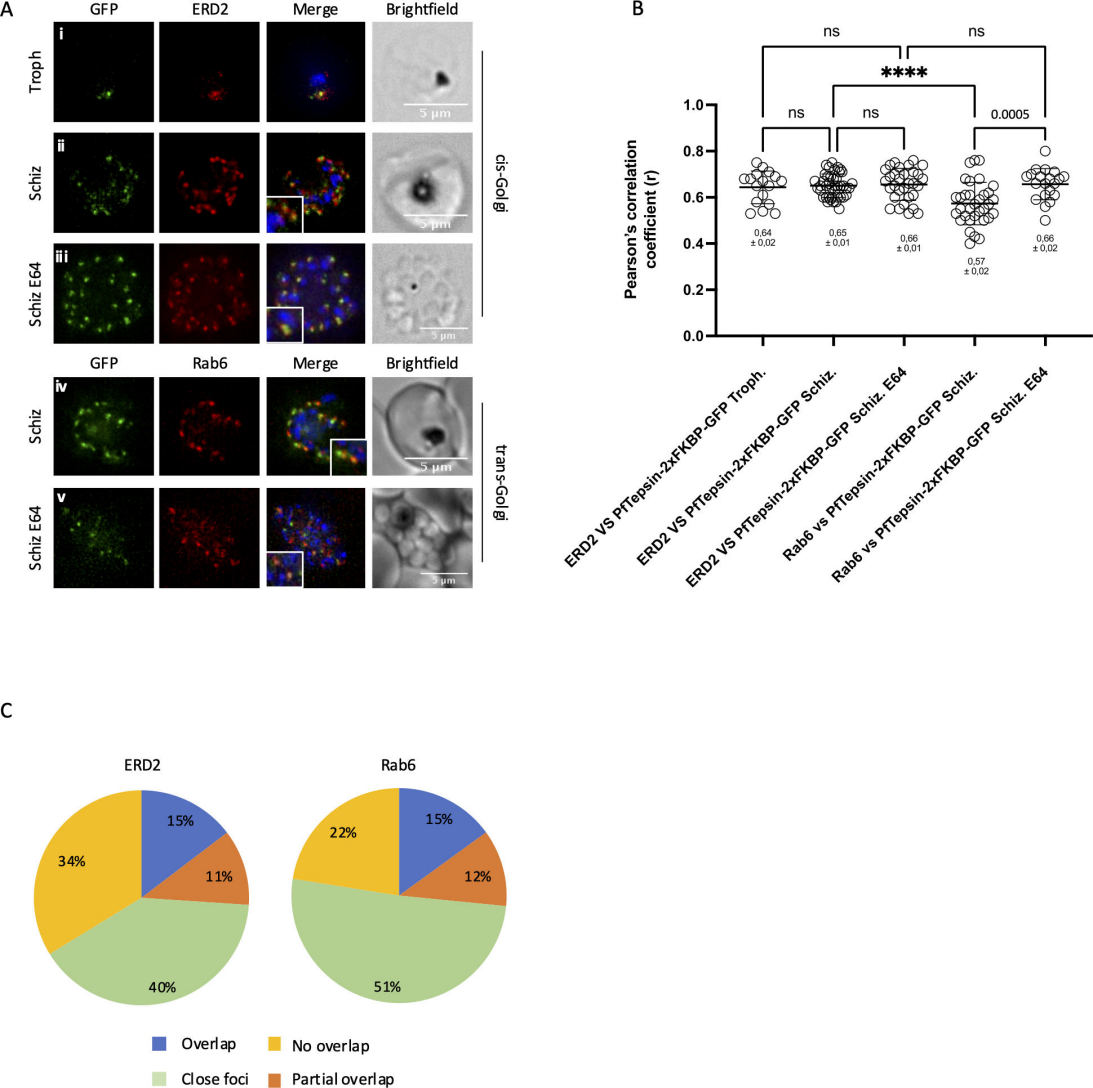
Colocalization analysis of PfTepsin with markers of the Golgi apparatus. Immunofluorescence assays to determine the overlap between PfTepsin-2xFKBP-GFP and the cis-Golgi marker ERD2 in trophozoites (Ai), developing schizonts (Aii) and late schizonts treated with E64 (Aiii); and with the trans-Golgi marker Rab6 in developing schizonts (Aiv) and late schizonts treated with E64 (Av). Scale bar represents 5 µm. Blue denotes the DAPI-stained nucleus. Schiz, developing schizonts; Troph, trophozoites. (**B**) Pearson’s correlation analysis demonstrates that PfTepsin-2xFKBP-GFP overlaps significantly more with ERD2 than Rab6 in developing schizonts but not in late schizonts treated with E64. PfTepsin-2xFKBP-GFP vs ERD2; Trophs, *n* = 17; developing schizonts, *n* = 42; E64 schizonts, *n* = 31. PfTepsin-2xFKBP-GFP vs Rab6; developing schizonts, *n* = 33; E64 schizonts, *n* = 20. Data pooled from at least three biological replicates. Values represent the mean ± standard error. *P* values were calculated using an unpaired *t*-test. ****P < 0.0001. ns, >0.9000. (**C**) Quantification of the foci distribution in individual merozoites in developing schizonts. Overlap in blue, partial overlap in orange, close foci in green, and no overlap in yellow. Raw data available in supplementary file named Colocalization counts.

In order to determine the subcellular localization of PfTepsin, colocalization assays with markers of different organelles were performed. Since Tepsins are accessory trafficking proteins of the AP-4 complex (45) and the vesicles coated by AP-4 are originating from the trans-Golgi network in mammalian cells (51, 52), we wanted to determine whether PfTepsin was found at the Golgi apparatus. Indeed, some partial overlap between PfTepsin-2xFKBP-GFP and the cis- and trans-Golgi markers ERD2 (53) and Rab6 (54) could be seen (Fig. 3A). Quantification of the level of colocalization was done by Pearson’s correlation analysis and revealed that the coefficient value for PfTepsin-2xFKBP-GFP vs Rab6 was lower than that for ERD2 (0.57 ± 0.02 and 0.65 ± 0.01, respectively) (Fig. 3B). Closer inspection of individual merozoites inside schizonts showed that for both ERD2 and Rab6, the level of overlap with PfTepsin was quite varied, so we classified them into four categories: no colocalization when there was no overlap between the red and green signals, close foci when red and green signals were very close to each other (=less than one focus diameter apart) but did not overlap, partial colocalization when some pixels from one green focus overlapped with pixels from a red focus, and finally, overlap when the foci extensively overlapped each other. The proportion of ERD2 or Rab6 foci that fully or partially colocalized with PfTepsin was similar for the two markers, with values between 12% and 15%. 40% of the ERD2 signals were close to PfTepsin and 51% for Rab6. Finally, 34% of the ERD2 foci did not overlap at all with PfTepsin and 22% for Rab6 (Fig. 3C). In several types of eukaryotic cells, the Golgi apparatus is stacked with well-differentiated cisternae performing distinct functions (55, 56); however, in *P. falciparum*, extensive Golgi stacks have not been observed (57, 58). Instead, markers of the cis- and trans-Golgi partially colocalize in rings and trophozoite stages and become more segregated as schizogony progresses (54). We therefore decided to investigate the localization of PfTepsin in very late schizonts where egress was blocked by the protease inhibitor E64. Interestingly, the level of overlap of PfTepsin with Rab6 increased in late schizonts, and there was no longer a difference with ERD2 (0.66 ± 0.02 and 0.65 ± 0.01, respectively) (Fig. 3B). Taken together, these results seem to suggest that PfTepsin might be transiting through both domains of the Golgi. Recent work on the Tepsin homolog of the related apicomplexan *Toxoplasma gondii* has shown that it was likely localizing at the trans-Golgi since the fluorescence signal was downstream of the cis-medial Golgi marker GRASP55 (59). Conditional knockdown of TgTepsin resulted in parasite death due to major disruptions of the IMC, which led the authors to speculate that the protein might be involved in vesicular trafficking between the trans-Golgi and the IMC (59).

**Fig 4:**
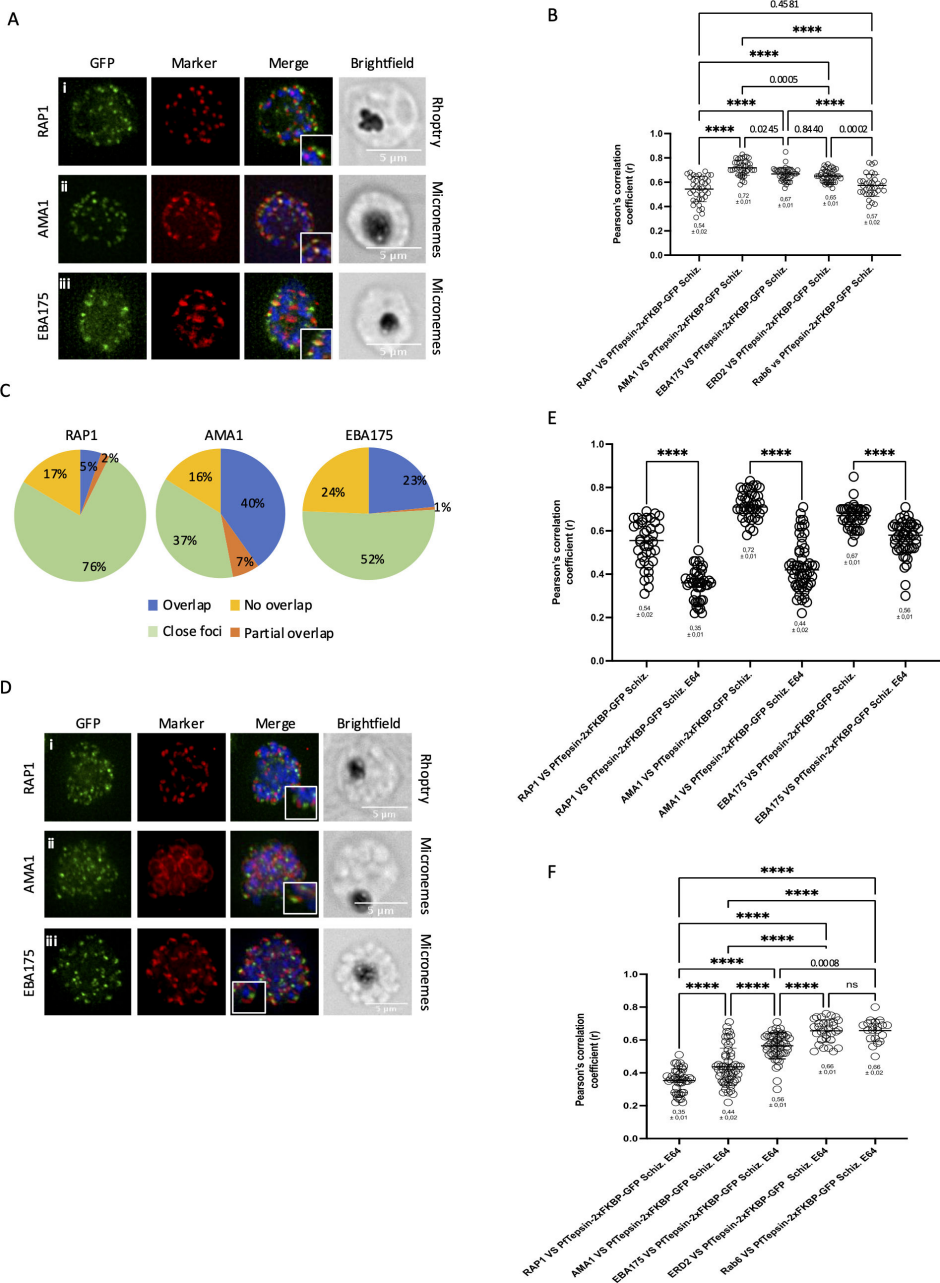
Colocalization analysis of PfTepsin with markers of the apical complex organelles. (**A**) Immunofluorescence assays on developing schizont stage parasites to determine the overlap between PfTepsin-2xFKBP-GFP and (Ai) the rhoptry marker RAP1, (Aii) the microneme marker AMA1, and (Aiii) the microneme marker EBA175. PfTepsin-2xFKBP-GFP vs RAP1, *n* = 38; PfTepsin-2xFKBP-GFP vs AMA1, *n* = 39; PfTepsin-2xFKBP-GFP vs EBA175, *n* = 38; Scale bar represents 5 µm. Blue denotes the DAPI-stained nucleus. (**B**) Pearson’s correlation analysis demonstrates that PfTepsin-2xFKBP-GFP overlaps significantly more with AMA1 than EBA175 and RAP1 in developing schizonts. Data pooled from at least three biological replicates. Data for the Golgi markers taken from Fig. 3B. Values represent the mean ± standard error. *P* values were calculated using one-way analysis of variance (ANOVA) followed by Tukey’s multiple comparison test. ****P < 0.0001. (**C**) Quantification of the foci distribution between PfTepsin-2xFKBP-GFP and AMA1 and EBA175 in individual merozoites of developing schizonts. Overlap in blue, partial overlap in orange, close foci in green, and no overlap in yellow. Raw data available in supplementary file named Colocalization counts. (**D**) Immunofluorescence assays on late schizont stage parasites treated with E64 to determine the overlap between PfTepsin-2xFKBP-GFP and (Di) the rhoptry marker RAP1, (Dii) the microneme marker AMA1, and (Diii) the microneme marker EBA175. PfTepsin-2xFKBP-GFP vs RAP1, *n* = 42; PfTepsin-2xFKBP-GFP vs AMA1, *n* = 55; PfTepsin-2xFKBP-GFP vs EBA175, *n* = 49; Scale bar represents 5 µm. Blue denotes the DAPI-stained nucleus. (**E**) Pearson’s correlation analysis demonstrates that PfTepsin-2xFKBP-GFP overlaps significantly less with the markers of the apical complex in late schizonts treated with E64 than in developing schizonts. Data pooled from at least three biological replicates. Values represent the mean ± standard error. *P* values were calculated using one-way ANOVA followed by Tukey’s multiple comparison test. ****P < 0.0001. (**F**) Statistical analysis showing that in late schizonts treated with E64, PfTepsin-2xFKBP-GFP overlaps more with the markers of the Golgi apparatus than with any of the apical complex markers. Data for the Golgi markers taken from Fig. 3B. ****P < 0.0001; ns, *P* > 0.9999.

We next wanted to see if PfTepsin localized to the apical complex in developing schizonts using antibodies targeting the rhoptry marker RAP1 (60) and the microneme markers AMA1 (61) and EBA175 (62) in immunofluorescence assays. Upon visual inspection, it seemed that limited colocalization was observed with RAP1, while variable levels of overlap were seen with both microneme markers (Fig. 4A). Quantification by Pearson's correlation analysis gave a coefficient of 0.54 ± 0.02 for RAP1 vs PfTepsin-2xFKBP-GFP, 0.72 ± 0.01 for AMA1 vs PfTepsin, and 0.67 ± 0.01 for EBA175 vs PfTepsin. Statistical analysis revealed that PfTepsin was overlapping more significantly with the microneme markers than with the rhoptry marker. Comparison with the Golgi markers was more variable. While the overlap between PfTepsin and AMA1 was higher than either ERD2 or Rab6, the overlap between PfTepsin and EBA175 was only higher for Rab6 but similar for ERD2. The reverse situation was found for RAP1 (Fig. 4B). Like what we had observed with the Golgi markers, the levels of overlap were quite different between individual merozoites in developing schizonts. Quantification showed that 5% of the PfTepsin foci overlapped with RAP1, 40% with AMA1, and 23% with EBA175. Partial overlap of 2% was seen with RAP1, 10% with AMA1, and 1% with EBA175. %76 of the RAP1 foci were observed close to PfTepsin, 37% for AMA1, and 52% for EBA175. Finally, 17% of foci did not overlap with RAP1, 16% with AMA1, and 24% with EBA175 (Fig. 4C). When looking at late schizonts (incubated with E64), a decrease in the colocalization of PfTepsin with all three markers was noted (Fig. 4D and E). Comparison with the Pearson correlation coefficients from the Golgi markers showed that at this stage, PfTepsin was colocalizing more with them than any of the apical complex markers (Fig. 4F). AMA1 and EBA175 reside in two different populations of micronemes, which are secreted at different times during the merozoite egress and invasion processes (8, 63, 64). Recent seminal work using expansion microscopy has shown that AMA1 forms puncta below the apical polar ring, while the EBA175 puncta are more basal, toward the rhoptry bulb (5). Taken together, the results of our analysis suggest that PfTepsin could potentially be trafficking between the Golgi and the organelles of the apical complex, which would be consistent with a putative role in vesicular trafficking between these cellular structures. Whether the protein cycles back and forth between the organelles or if the transit is only one way remains to be determined. The biogenesis of the apical complex and therefore the trafficking of proteins from the Golgi to its organelles are potentially much reduced in E64-treated schizonts since these are stalled in the process of rupturing, which might explain why PfTepsin does not colocalize as much when compared to developing schizonts.

### Clathrin and AP-4 complexes are potential interactors of PfTepsin

**Fig 5:**
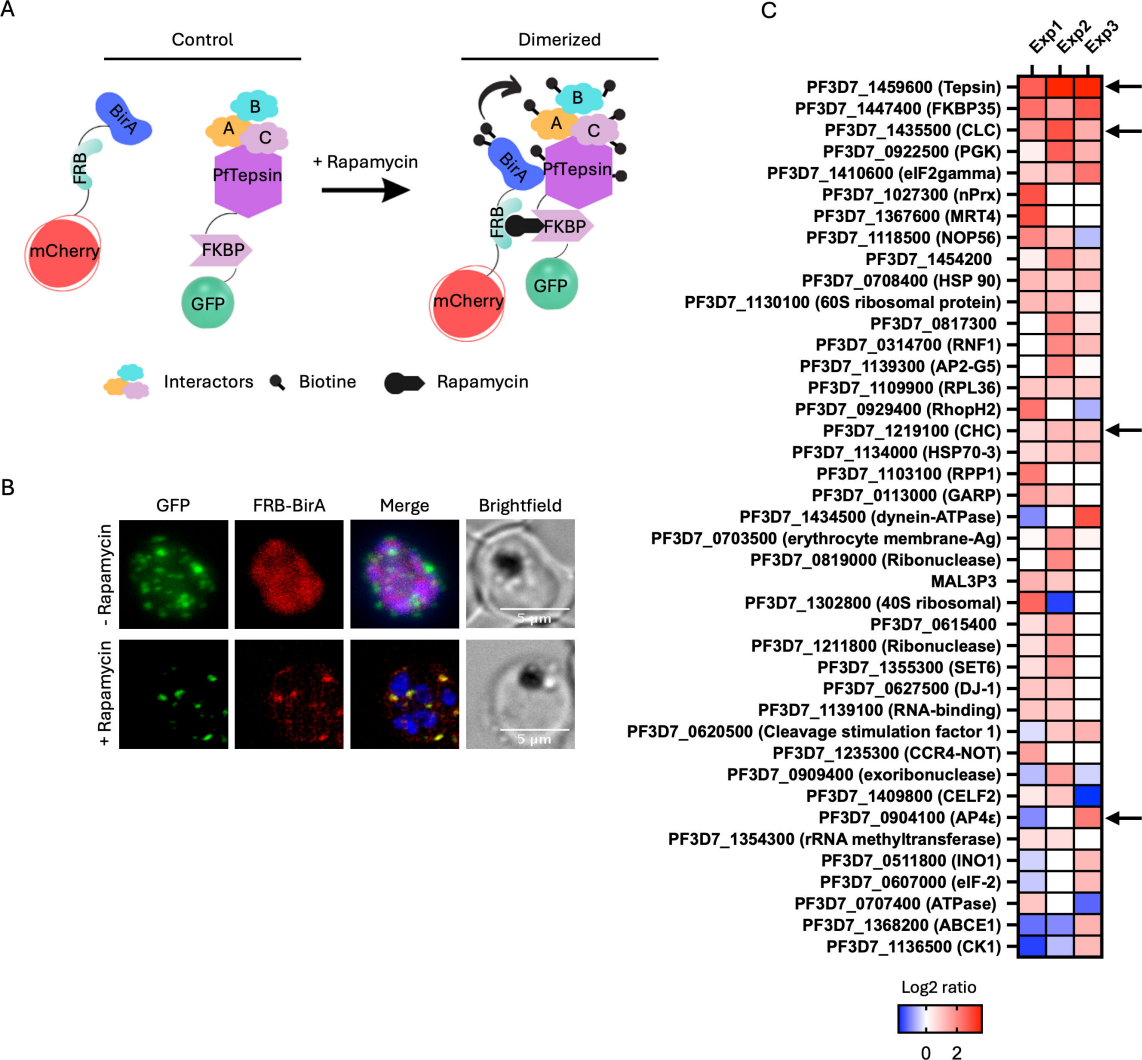
Identification of proteins proximal to PfTepsin by Di-BioID. (**A**) Schematic of the principle of Di-BioID. (**B**) Live microscopy showing that incubating the PfTepsin-2xFKBP-GFP + FRB-BirA-mCherry parasite line with 250 nM rapamycin for 24 h results in the translocation of the mCherry signal to the PfTepsin-2xFKBP-GFP foci. Scale bar represents 5 µm. Blue denotes the DAPI-stained nucleus. (**C**) Heat map showing the proteins enriched in each of the three biological replicates. Arrows highlight PfTepsin, clathrin heavy and light chains, and the AP-4 epsilon subunit. Complete data sets available in the supplementary document named IP-MSdata.

To try to gain more information on the function of PfTepsin, we utilized Di-BioID, a method allowing the identification of proximal proteins, some of which may be true interactors. For this, the PfTepsin-2xFKBP-GFP parasite line was transfected with a plasmid allowing the expression of an FRB domain fused to the BirA biotin ligase and mCherry. Upon addition of rapamycin and biotin to the culture medium, interaction between FKBP and FRB will lead to the biotinylation of proteins proximal to PfTepsin-2xFKBP-GFP(23) (Fig. 5A). Rapamycin and biotin were added in late rings for 24 h, and the parasites were harvested at the schizont stage. In parasites without Rapa, the FRB-BirA-mCherry signal is dispersed in the cytoplasm; however, addition of Rapa leads to its colocalization with PfTepsin-2xFBKP-GFP (Fig. 5B). Precipitation of biotinylated proteins with streptavidin-agarose beads and their identification by mass spectrometry showed that PfTepsin was the top hit, confirming that the FRB-BirA was properly recruited to the target protein (Fig. 5C). More than 300 proteins were identified in each of the three independent biological replicates. To refine the results, we initially considered only proteins with a minimum of five peptides and a peptide threshold of 95%. With these conditions, the number of hits decreased to 138 proteins. For each protein, the ratio of Rapa+/Rapa- control was calculated, and we retained only proteins with a ratio greater than 2. This led to the identification of around 50 potential interacting partners. Closer inspection showed that most were contaminants often found in IP-MS experiments, such as heat shock and ribosomal proteins. Interestingly, the light and heavy chains of clathrin and the epsilon subunit of the AP-4 complex were identified as putative interactors, though this would have to be confirmed by direct immunoprecipitation. In mammalian cells, AP-4 is recruited to the trans-Golgi by the small GTPase Arf1 (65, 66), where it is implicated in the sorting of specific protein cargoes to an endosomal compartment and potentially the plasma membrane (65, 67–69). Tepsin is an accessory protein of AP-4 with which it interacts via the ear domains of the AP-4 subunits ε and β (70, 71). It is proposed that Tepsin plays a role in the formation of AP-4-containing vesicles at the trans-Golgi (70). At steady state, AP-4 and Tepsin are in separate cellular pools in mammalian cells and potentially only interact during vesicle formation (45). This might potentially explain why we identified AP-4 in only one of the three biological replicates (Fig. 5C). Interestingly, in organisms where AP-4 is lost, such as yeast, worms, and flies, Tepsin is also absent (71).

The identification of clathrin as a potential partner of PfTepsin was surprising since most of the studies on AP-4 and Tepsin in mammalian cells report no interaction of these two proteins with clathrin. For example, none of the AP-4 subunits are able to bind to clathrin, and AP-4 is also not found in purified clathrin-coated vesicles (65, 72). A consensus clathrin-binding motif was likewise not found in human Tepsin (73). However, one report showed colocalization between AP-4 and the clathrin coat machinery by immunoelectron microscopy on Madin–Darby canine kidney cells (74). An interaction between AP-4, Tepsin, and clathrin was also detected in the protozoan parasite *Trypanosoma cruzi (75*). Further support for the interaction between Tepsin and clathrin in *P. falciparum* is provided by two independent studies examining the PfClathrin interactome/proxiome (23, 41). In addition, a motif search on the Eukaryotic Linear Motif Resource website (http://elm.eu.org/index.html) revealed two clathrin box motifs on PfTepsin (AA: 506–510 and 658–662). In eukaryotic cells, clathrins have roles in multiple processes such as in clathrin-mediated endocytosis, cargo recognition, and membrane trafficking (76, 77). It localizes to the plasma membrane and to the trans-Golgi and is recruited by AP-1 and AP-2 (78, 79). In *T. gondii*, clathrin was localized to post-Golgi structures and shown to be implicated in vesicular transport to the IMC, plasma membrane, micronemes, and rhoptries, with no evidence for a role in endocytosis (80). Moreover, TgClathrin was found to interact with AP-1 (81). In *P. falciparum*, the localization and the role of clathrin are still unclear. It was recently shown that although AP-2 is localized to the cytostome and is implicated in endocytosis of host-cell cytosol, PfClathrin was not (23, 41). PfClathrin was found to interact with PfAP-1, the latter being potentially implicated in the trafficking of proteins to the rhoptry (42).

To assess potential roles of PfTepsin in asexual erythrocytic stages, we attempted to mislocalize the protein to the nucleus by knock-sideways (50). For this, the PfTepsin-2xFKBP-GFP line was transfected with a mislocalizer containing three nuclear localization signals fused to FRB and mCherry. In the absence of rapamycin, the mislocalizer nicely overlaps with the DAPI-stained nucleus (Fig. S2A). However, upon visual inspection, the addition of rapamycin did not seem to cause much translocation of PfTepsin-2xFKBP-GFP to the nucleus. Quantification by Pearson's correlation analysis revealed that the addition of Rapa increased the overlap between the DAPI-stained nucleus and GFP and also between the mCherry-tagged mislocalizer and GFP, but had no impact on the overlap between DAPI and mCherry (Fig. S2B). This means that there is some translocation of PfTepsin to the nucleus, even though it is not obvious by eye. We next performed growth assays but did not see any difference in parasite proliferation in the presence of Rapa between the control pSLI-PfTepsin-2xFKBP-GFP strain without mislocalizer and the one with the mislocalizer (Fig. S2C). We do not know whether the absence of a growth defect is due to insufficient translocation or the non-essentiality of PfTepsin. However, our previous inability to inactivate its gene by SLI (24) and the low mutagenesis score from the whole-genome piggyback screen (82) suggest that it is potentially essential. Perhaps a mislocalizer targeting the plasma membrane or a sandwich version containing four FKBP domains instead of two would have been more suitable (50) or other methods of conditional regulation such as the glmS ribozyme (83) and the TetR-DOZI system (84).

### Conclusion

In conclusion, we present evidence for the presence of a homolog of PfTepsin in *P. falciparum* that is expressed throughout the asexual erythrocytic cycle and potentially binds phosphoinositides. Our colocalization analyses suggest that the protein might be trafficking between the Golgi apparatus and some of the organelles of the apical complex. Finally, we provide evidence that PfTepsin potentially interacts with the clathrin and AP-4 complexes.

## MATERIALS AND METHODS

### Protein structure analysis

Protein domain alignment was performed using UCSF ChimeraX (85). The individual domains were identified based on their predicted aligned error scores from AlphaFold (44). Each domain was then independently aligned to the reference structure using the MatchMaker tool, which also provides rmsd. The electrostatic surface analysis was conducted using the “color surface by electrostatic potential” tool from UCSF ChimeraX. The electrostatic potential was calculated using the Coulombic surface coloring method, which applies Coulomb’s law: Φ=Σ[qi / *ϵd*i], where Φ is the potential; *q* is the atomic partial charge; *d* is the distance from the atoms; and *ϵ* is the dielectric constant. The electrostatic potential was mapped onto the molecular surface using a color gradient from red (negative potential) to white (neutral) to blue (positive potential).

### Production and purification of the recombinant PfTepsin ENTH domain

The ENTH domain of PfTepsin (amino acids 7–125) was amplified on 3D7 WT mixed-stage cDNA using primers 5′BamH1‐PftENTHdomain (ATAGGATCCATGATGAATAGGTTAATTTTGAAC) and 3′Xho1‐PftENTHdomain (ATACTCGAGTTTCATAACATTTTCTGTAGC) and cloned in pGEX‐6P3 (GE Healthcare) to be expressed as a recombinant GST fusion protein. *Escherichia coli* BL21 (DE3) competent cells were transformed with the pGEX-6P3-PfTepsin-tENTH domain. Before the induction of the fusion protein, transformed cells were grown at 37°C until the OD_600_ reached 0.4–0.6. Then, the fusion protein was induced with 0.3 mM isopropyl β‐D‐1‐thiogalactopyranoside for 3 h at 37°C. Bacterial pellets were resuspended in lysis buffer (phosphate-buffered saline [PBS], pH 7.4 + 1 mM EDTA + 1× protease inhibitor cocktail (Sigma-Aldrich) and frozen. Subsequently, cells were thawed, and fresh lysozyme at 10 mg/mL was added, as well as dithiothreitol (DTT) at 5 mM. Cells were sonicated four times for 30 s on ice. Protein solubilization was performed with 1% Triton X‐100 for 10 min at room temperature (RT), then centrifuged at 30,000 × *g* for 25 min at 4°C. Proteins were purified with glutathione agarose (Sigma) according to the manufacturer’s instructions, eluted with elution buffer (20 mM Tris; 150 mM NaCl; 20 mM reduced glutathione, pH 9.0), and analyzed by SDS-PAGE, and Coomassie staining was performed for purity check.

### Protein–lipid overlay assay

The lipid overlay assay was performed using PIP Strips (Echelon Biosciences). Briefly, the PIP strips were blocked overnight with PBS-Tween (PBS-T) 0.1% buffer + 3% bovine serum albumin lipid free at 4°C. After three washes with PBS-T 0.1% buffer, the membrane was incubated with 2 µg/mL of recombinant proteins [GST alone, PI(4,5)P2-binding PlcD-PH domain fused to GST and GST‐PfTepsin tENTH domain] for 1 h at room temperature. After three washes with the same buffer, the membranes were incubated for 1 h at room temperature with anti‐GST antibody (1:5,000; Bethyl Laboratories). After three more washes with the same buffer, the membranes were incubated for 1 h at room temperature with horseradish peroxidase (HRP)‐conjugated anti‐rabbit antibody (1:10,000; Abcam) followed by three washes. The bound proteins were detected with the Clarity Western ECL kit from Bio‐Rad Laboratories.

### Parasite culture

*P. falciparum* 3D7 asexual stage parasites were obtained from David Walliker, Edinburgh University. The parasites were maintained under standard conditions in Roswell Park Memorial Institute-HEPES medium at 4% hematocrit (human erythrocytes of O + group) and 0.5% (wt/vol) Albumax (Invitrogen) and kept at 37°C in a gas mixture of 5.0% oxygen, 5.0% carbon dioxide, and 90% nitrogen (86).

### Vector constructions and transfection

To endogenously tag PfTepsin with 2xFKBP-GFP, we used the selection-linked integration strategy (50). Around 500 bp of the N-terminus of *PfTepsin* was amplified with primers 5′Not1-Pf3D7-1459600 (ATAGCGGCCGCAATAAAAATAATATGAATAAGAATTATAATAATAATG) and 3′AvrII-stopless-Pf3D7-1459600 (ATACCTAGGTAGTTTCATATGATCCGATAATAG), digested and cloned in frame with 2xFKBP-GFP in pSLI-2xFKBP-GFP digested NotI-AvrII. Parasites were transfected, and integrants were selected as described previously with some modifications (50). Briefly, *P. falciparum* 3D7 parasites were transfected with 100 µg of the pSLI-PfTepsin-2xFKBP-GP plasmid. A first positive selection for transfectants was performed using 5 nM WR99210 (WR, Jacobus Pharmaceuticals). Drug-resistant parasites were split into three separate wells with 1%–2% parasitemia and went under a second round of selection with 400 mg/mL neomycin to select for integrants. After parasite reemergence (after around 10 days), WR was put back in the culture medium. Proper integration was verified by PCR. For the integration in 5′, a gene of interest (GOI)-specific forward primer 1 (GCTTAAATGTTAAGGGTAATAACACC), along with primer 2: FKBP-sandwich-rev (CAGAGCAGCTCTAGCAGC), was used. For the 3′ integration event, primer 3: M13_rev (CAGGAAACAGCTATGAC) and a GOI-specific reverse primer 4 were used (CATACTATAAAACAAGGAAATATAATATACAC).

To generate the pHSP86p-mScarlet-Linker-Rab6-DHODH plasmid, the coding sequence of the *Rab6* gene was amplified by PCR from 3D7 cDNA using 5′ Mlu1-Rab6 (ATAACGCGTATGGATGAATTTCAAAACTC) and 3′ XhoI-Rab6 (ATACTCGAGTTAACATAAACATTTACTTAACATATTTTTG). Double transfectants were generated by transfecting 100 µg of pHSP86p-mScarlet-Linker-Rab6 in the pSLI-PfTepsin-2xFKBP-GFP line. Selection of transfectants was done with 0.9 µM of DSM1 (BEI Resources).

### Western blotting

To verify the expression of PfTepsin-2xFKBP-GFP, mixed-stage parasites were harvested by saponin lysis, and the pellet was solubilized in SDS protein sample buffer. Proteins were separated on 7% (wt/vol) SDS-polyacrylamide gel under reducing conditions and transferred to a polyvinylidene difluoride membrane (Millipore). The blocking was done with 4% (wt/vol) milk in Tris-buffered saline with Tween 20 for 30 min at RT. The membrane was first incubated with a mouse monoclonal anti-GFP (clone JL-8, Roche) (diluted 1:1,000) for 1 h at RT and with mouse HRP-coupled secondary antibodies (Sigma) (diluted 1:10,000) for 30 min at RT. Immunoblots were developed using ECL (Bio-Rad).

### Fluorescence microscopy

Fluorescence images of parasites were captured using a GE Applied Precision Deltavision Elite microscope with ×100 1.4 NA objective and with a sCMOS camera and deconvolved with the SoftWorx software. Chromatic calibration of the microscope was performed prior to imaging experiments. For immunofluorescence assays, parasites were fixed on pre-treated coverslips with 0.01% vol/vol poly-L-lysine using 4% paraformaldehyde (ProSciTech) (87). Then, parasites were permeabilized with 0.1% Triton X-100 (Sigma-Aldrich). Blocking was made with 3% bovine serum albumin (Sigma Aldrich) during 1 h followed by incubation for 1 h with primary antibodies: rabbit polyclonal anti-PfERD2 (1:2,000) (53), mouse monoclonal anti-RAP1 (1:2,000) (60), mouse monoclonal anti-AMA1 (clone 1F9; 1:1,000) (61), and rabbit anti-PfEBA175 (1:1,000) (62). Bounded antibodies were then visualized with either Alexa Fluor-594 antirabbit or antimouse diluted 1:1,000 (Cedarlane). Parasites were mounted in Vectashield (Vecta Laboratories) containing 0.1 mg/mL DAPI (Invitrogen).

For live-cell imaging, PfTepsin-2xFKBP-GFP parasites and the cotransfected lines were incubated with DAPI for 10 min prior to visualizing them. Images shown represent a single optical slice from a deconvolved z-stack.

Pearson’s correlation coefficients were calculated on deconvolved regions of interest of image stacks, including zero–zero pixels and without thresholding using the SoftWorx software (GE). Data were analyzed for statistical significance using either an unpaired *t*-test or one-way analysis of variance followed by Tukey’s multiple comparison test. Chromatic calibration of the microscope was performed prior to imaging experiments. For the quantification of the overlap, around 20 parasites from the Pearson's correlation analyses were randomly selected. The experimental conditions were blinded to the person performing the analysis.

### Preparation of samples for Di-BioID

Preliminary to the Di-BioID experiment, PfTepsin-2xFKBP-GFP + FRB-BirA-mCherry parasites were tightly synchronized, and late rings were grown with 50 µM biotin (Sigma-Aldrich) and with or without 250 nM rapamycin (R0395, Sigma-Aldrich) for 20–24 h. Dimerization between the bait protein and BirA was confirmed in schizont-stage parasites by live-cell microscopy. The Di-BioID experiments were performed as previously described (23). After the 20–24 h incubation, parasites were harvested and centrifuged at 1,200 rpm, washed twice in PBS before being saponin-lysed. Saponin pellets were frozen at −80°C. The day after, pellets were thawed and resuspended in 2 mL of cold lysis buffer (50  mM Tris-HCl, pH 7.5, 500 nM NaCl, 1% Triton X-100 (Sigma-Aldrich), 1 mM DTT (Sigma-Aldrich), 1 mM phenylmethylsulfonyl fluoride (Sigma-Aldrich) with CØmplete EDTA-free protease inhibitor cocktail tablet (Roche). After two freeze–thaw cycles, cell disruption was performed by three rounds of sonication (model 100, Fisher Scientific) for 10 s with 30 s breaks. Samples were centrifuged at 16,000 × *g*, and supernatants were incubated with streptavidin agarose beads (Invitrogen) at 4°C O/N to enrich biotinylated proteins. The next day, the beads were washed twice with lysis buffer, once in dH_2_O, twice in Tris-HCL (pH 7.5), and finally, five times in 50 mM ammonium bicarbonate (Sigma-Aldrich).

### Sample preparation and data acquisition for mass spectrometry analysis

Protein digestion and mass spectrometry experiments were performed by the Proteomics platform of the CHU de Quebec Research Center, Quebec, Canada.

### Protein digestion

Protein digestion and mass spectrometry experiments were performed by the Proteomics platform of the CHU de Quebec Research Center. On beads, protein digestion was carried out using 0.1 µg of modified porcine trypsin (sequencing grade; Promega, Madison, WI, USA) in 50 mM ammonium bicarbonate for 5 h at 37°C. Digestion was stopped with 5% formic acid (FA), and peptides were eluted from the beads with 60% acetonitrile (ACN) and 0.1% FA. Tryptic peptides were desalted on stage tips (Empore C18, 3M Company), vacuum dried, then resuspended in LC loading solvent (2% ACN, 0.05% trifluoroacetic acid [TFA]).

### Mass spectrometry

Half of each sample was analyzed by nanoLC/tandem mass spectrometry (MS/MS) using a Dionex UltiMate 3000 nanoRSLC chromatography system (Thermo Fisher Scientific, San Jose, CA, USA) connected to an Orbitrap Fusion mass spectrometer (Thermo Fisher Scientific) equipped with a nanoelectrospray ion source. Peptides were trapped at 20 µL/min in loading solvent (2% ACN, 0.05% TFA) on a 5 mm × 300 µm C18 pepmap cartridge (Thermo Fisher Scientific) for 5 min. Then, the pre-column was switched online with a 50 cm × 75 µm internal diameter separation column (Pepmap Acclaim column, Thermo Fisher Scientific), and the peptides were eluted with a linear gradient from 5% to 40% solvent B (A: 0.1% FA, B: 80% ACN, 0.1% FA) for 30 min, at 300 nL/min (60 min total runtime). Mass spectra were acquired using a data-dependent acquisition mode using Thermo XCalibur software (version 4.1.50). Full scan mass spectra (350–1,800 m/z) were acquired in the Orbitrap using an AGC target of 4e5, a maximum injection time of 50 ms, and a resolution of 120,000. Internal calibration using lock mass on the m/z 445.12003 siloxane ion was used. Each MS scan was followed by the acquisition of fragmentation MS/MS spectra of the most intense ions for a total cycle time of 3 s (top speed mode). The selected ions were isolated using the quadrupole analyzer with 1.6 m/z windows and fragmented by higher-energy collision-induced dissociation with 35% of collision energy. The resulting fragments were detected by the linear ion trap in rapid scan rate with an AGC target of 1e4 and a maximum injection time of 50 ms. Dynamic exclusion of previously fragmented peptides was set for a period of 30 s and a tolerance of 10 ppm.

### Database searching

MGF peak list files were created using Proteome Discoverer software (version 2.3, Thermo Fisher Scientific). MGF files were then analyzed using Mascot (version 2.8.0; Matrix Science, London, UK). Mascot was set up to search a contaminant database and Uniprot Plasmodium Falciparum 3D7 (5538 entries, reference proteome UP000001450) database, assuming the digestion enzyme trypsin. Mascot was searched with a fragment ion mass tolerance of 0.60 Da and a parent ion tolerance of 10 ppm. Carbamidomethyl of cysteine was specified in Mascot as a fixed modification. Deamidation of asparagine and glutamine and oxidation of methionine were specified in Mascot as variable modifications. Two missed cleavages were allowed.

### Criteria for protein identification

Scaffold (version Scaffold_5.1; Proteome Software Inc., Portland, OR, USA) was used to validate MS/MS-based peptide and protein identifications. A false discovery rate of 1% was used for peptide and protein. Proteins that contained similar peptides and could not be differentiated based on MS/MS analysis alone were grouped to satisfy the principles of parsimony.

### Knock sideways attempts

Tightly synchronous ring-stage PfTepsin2xFKBP-GFP + mislocalizer parasites were seeded at 2% parasitemia and grown with ±250 nM rapamycin. Once they reached the schizont stage, the cells were harvested, stained with DAPI (100 ng/μL, Invitrogen) and imaged immediately. For the growth assays, parasite cultures of PfTepsin-2xFKBP-GFP with and without mislocalizer were seeded at about 0.1% parasitemia, and each received 250 nM rapamycin. After 24, 72, and 120 h in culture, parasites were sampled and analyzed by fluorescence-activated cell sorting (FACS) on a BD FACSCanto to evaluate parasitemia as described in references 9 and 88. Summarily, the parasites were stained with SYBR Gold (Invitrogen-Molecular Probe), followed by fixation with 1% paraformaldehyde for 1 h. A total of 100,000 events were recorded on the FACSCanto A using the FACSDiva software, and the results were analyzed using the FlowJo software. Uninfected red blood cells were used to determine the fluorescein isothiocyanate signal threshold.

## Data Availability

The data supporting the findings of this study are available within the paper and are also available from the corresponding author upon request.

## SUPPLEMENTAL MATERIAL

Data S1 spectrum.03288-24-s0001.xlsx Raw values related to Fig. 3C and 4C.

Data S2 spectrum.03288-24-s0002.xlsx Complete data from the immunoprecipitation-mass spectrometry experiments. Related to Fig. 5.

Supplemental material spectrum.03288-24-s0003.pdf Fig. S1 and S2.

ASM does not own the copyrights to Supplemental Material that may be linked to, or accessed through, an article. The authors have granted ASM a non-exclusive, world-wide license to publish the Supplemental Material files. Please contact the corresponding author directly for reuse.
