# Supplementary material for: Characterization of the malaria parasite Plasmodium falciparum Tepsin homolog
Source: Microbiol Spectr. 2025 Jun 30;13(8):e03288-24. doi: 10.1128/spectrum.03288-24 (PMC12323583; doi:10.1128/spectrum.03288-24)
Supplement: Supplemental material — Fig. S1 and S2. [file spectrum.03288-24-s0003.pdf]

SUPPLEMENTARY DOCUMENTS TITLES

Figure S1. Additional microscopy images of PfTepsins-2xFKBP-GFP. Related to figure 2D. (A) Rings. (B) Trophozoites. (C) Schizonts.

Figure S2: Knock-sideways attempts. (A) Live cell microscopy showing that the addition of rapamycin does not result in a visibly strong translocation of the PfTepsin-2xFKBP-GFP to the nucleus. (B) Pearson's correlation analysis demonstrates that adding rapamycin increases the overlap of PfTepsin-2xFKBP-GFP with the Dapi-stained nucleus and with the 3xNLS-FRB-mCherry mislocalizer. Values represent the mean ± standard error. *P*-values were calculated using an unpaired t-test. ****: $p<0.0001$. Scale bar represents 5µm. Blue: DAPI stained nucleus.

Document named Colocalization counts: Raw values related to figures 3C and 4C

Document named IP-MSdata: Complete data from the immunoprecipitation-mass spectrometry experiments. Related to Fig. 5

Fig. S1

A

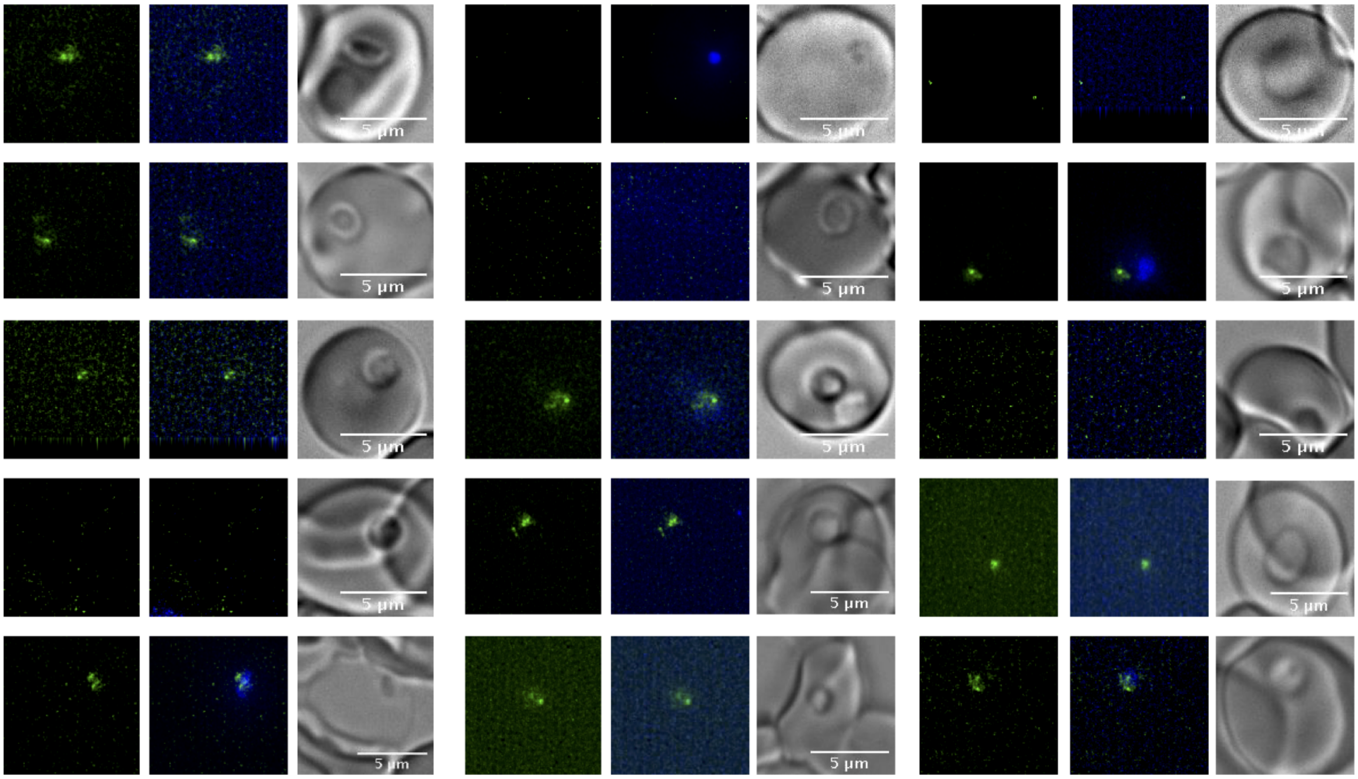

B

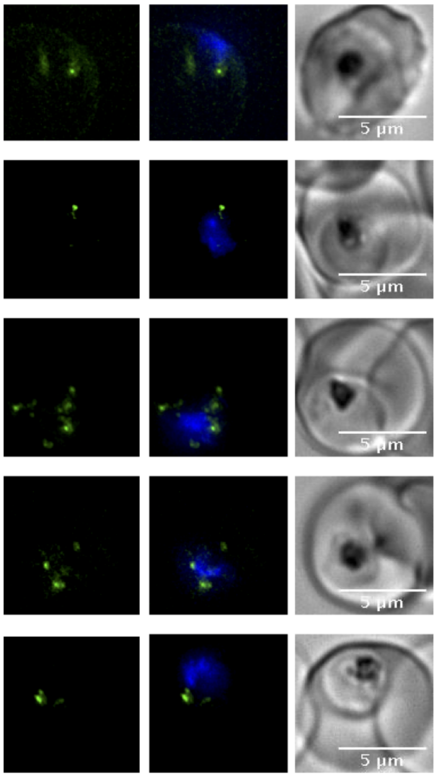

C

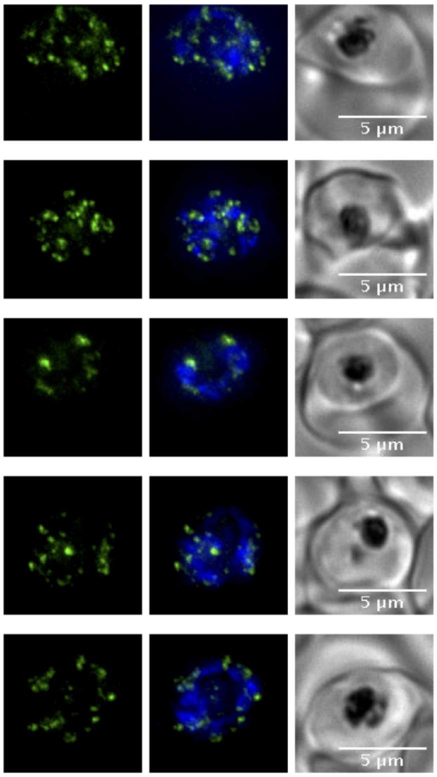

Fig. S2

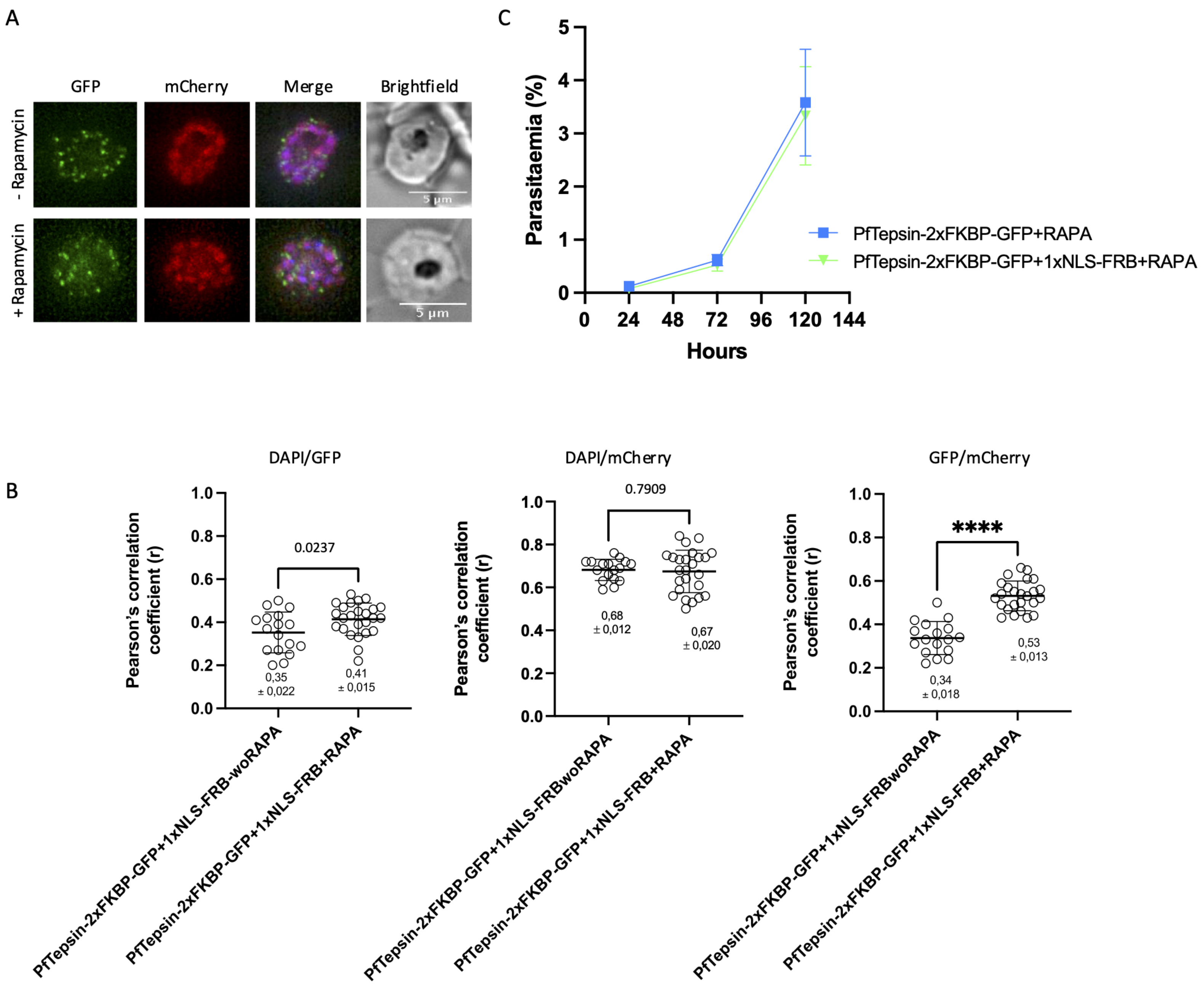

A
GFP
mCherry
Merge
Brightfield
- Rapamycin
+ Rapamycin
5 µm
5 µm
C
Parasitaemia (%)
5
4
3
2
1
0
0
24
48
72
96
120
144
Hours
PfTepsin-2xFKBP-GFP+RAPA
PfTepsin-2xFKBP-GFP+1xNLS-FRB+RAPA
B
DAPI/GFP
Pearson's correlation coefficient (r)
1.0
0.8
0.6
0.4
0.2
0.0
0.0237
0,35
± 0,022
0,41
± 0,015
PfTepsin-2xFKBP-GFP+1xNLS-FRB-woRAPA
PfTepsin-2xFKBP-GFP+1xNLS-FRB+RAPA
DAPI/mCherry
Pearson's correlation coefficient (r)
1.0
0.8
0.6
0.4
0.2
0.0
0.7909
0,68
± 0,012
0,67
± 0,020
PfTepsin-2xFKBP-GFP+1xNLS-FRBwoRAPA
PfTepsin-2xFKBP-GFP+1xNLS-FRB+RAPA
GFP/mCherry
Pearson's correlation coefficient (r)
1.0
0.8
0.6
0.4
0.2
0.0
****
0,34
± 0,018
0,53
± 0,013
PfTepsin-2xFKBP-GFP+1xNLS-FRBwoRAPA
PfTepsin-2xFKBP-GFP+1xNLS-FRB+RAPA
